# Combined proton radiography and irradiation for high-precision preclinical studies in small animals

**DOI:** 10.3389/fonc.2022.982417

**Published:** 2022-08-31

**Authors:** Moritz Schneider, Elisabeth Bodenstein, Johanna Bock, Antje Dietrich, Sebastian Gantz, Lena Heuchel, Mechthild Krause, Armin Lühr, Cläre von Neubeck, Sindi Nexhipi, Michael Schürer, Falk Tillner, Elke Beyreuther, Theresa Suckert, Johannes Richard Müller

**Affiliations:** ^1^ OncoRay, National Center for Radiation Research in Oncology- Faculty of Medicine and University Hospital Carl Gustav Carus- Technische Universitat Dresden-Helmholtz-Zentrum Dresden-Rossendorf, Dresden, Germany; ^2^ Helmholtz-Zentrum Dresden-Rossendorf, Institute of Radiation Physics, Dresden, Germany; ^3^ Helmholtz-Zentrum Dresden-Rossendorf, Institute of Radiooncology - OncoRay, Dresden, Germany; ^4^ German Cancer Consortium Deutsches Konsortium für Translationale Krebsforschung (DKTK), partner site Dresden- German Cancer Research Center DKFZ, Heidelberg, Germany; ^5^ Technical University (TU) Dortmund- Faculty of Physics, Medical Physics and Radiotherapy, Dortmund, Germany; ^6^ National Center for Tumor Diseases (NCT), Partner Site Dresden, Dresden, Germany; ^7^ Department of Radiotherapy and Radiation Oncology, Faculty of Medicine and University Hospital Carl Gustav Carus, Technische Universitat Dresden, Dresden, Germany; ^8^ Department of Particle Therapy, University Hospital Essen, University of Duisburg-Essen, Essen, Germany; ^9^ Deutsche Forschungsgemeinschaft Cluster of Excellence 'Physics of Life', Technische Universität Dresden, Dresden, Germany

**Keywords:** proton therapy, mouse brain irradiation, proton radiography, preclinical (*in vivo*) studies, DNA damage, relative biological effectiveness (RBE)

## Abstract

**Background and purpose:**

Proton therapy has become a popular treatment modality in the field of radiooncology due to higher spatial dose conformity compared to conventional radiotherapy, which holds the potential to spare normal tissue. Nevertheless, unresolved research questions, such as the much debated relative biological effectiveness (RBE) of protons, call for preclinical research, especially regarding *in vivo* studies. To mimic clinical workflows, high-precision small animal irradiation setups with image-guidance are needed.

**Material and methods:**

A preclinical experimental setup for small animal brain irradiation using proton radiographies was established to perform planning, repositioning, and irradiation of mice. The image quality of proton radiographies was optimized regarding the resolution, contrast-to-noise ratio (CNR), and minimal dose deposition in the animal. Subsequently, proof-of-concept histological analysis was conducted by staining for DNA double-strand breaks that were then correlated to the delivered dose.

**Results:**

The developed setup and workflow allow precise brain irradiation with a lateral target positioning accuracy of<0.26mm for *in vivo* experiments at minimal imaging dose of<23mGy per mouse. The custom-made software for image registration enables the fast and precise animal positioning at the beam with low observer-variability. DNA damage staining validated the successful positioning and irradiation of the mouse hippocampus.

**Conclusion:**

Proton radiography enables fast and effective high-precision lateral alignment of proton beam and target volume in mouse irradiation experiments with limited dose exposure. In the future, this will enable irradiation of larger animal cohorts as well as fractionated proton irradiation.

## 1 Introduction

Proton therapy is an increasingly used treatment modality for cancer patients (1). However, open research questions, such as the variable relative biological effectiveness (RBE) along the proton beam or novel therapeutics, call for preclinical experiments in a clinical like setting (2–4). The irradiation of organ subvolumes and orthotopic tumors in small animals offers high translational value (2, 5) and is in many aspects similar to clinical treatment, providing the right experimental conditions. Amongst others, researchers need to consider down-scaling of the target volumes, accurate small-field dosimetry, beam energies, and how to integrate appropriate imaging modalities into their workflow (6). Suitable equipment for irradiating small animals with protons that provides high positioning accuracy and clinical relevance is not available off-the-shelf and often needs to be custom-made. In recent years, several setups have been developed for small animal proton irradiation. The implemented solutions include visual positioning with digital cameras for image guidance (7), side illumination with a thin-foil mirror (8), and alignment lasers (9, 10). More advanced, but also cost-intensive, X-ray based commercial setups can be used to acquire CT images of small animals prior to the irradiation in order to precisely position the animal in the beam (11, 12). Technologically sophisticated ion-based tomographic imaging has been proposed for both animal studies and patient treatment (13–15). This imaging technique offers the advantage of using the same coordinate reference for imaging and irradiation as well as a precise stopping power estimation. 2D proton radiography is a basic form of ion radiography that uses a flat panel detector. This approach provides on-beam imaging, while being easy and cost-effective to implement. So far, it has been shown to be an effective means for setup verification (16); however, image quality has been deemed insufficient for small animal positioning  (17).

In this manuscript, we describe a workflow for the image-guided proton irradiation of small animals using proton radiography to image live mice during treatment positioning. The irradiation process incorporates the basic procedures of clinical therapy, i.e., planning, positioning, and irradiation of defined target volumes. High image resolution at low imaging dose is achieved by optimizing setup and imaging parameters. As proof-of-concept we overlaid a Monte Carlo simulation of the applied dose and the immunohistochemically quantified occurrence of DNA double-strand breaks. We use this method to demonstrate the induction of biological damage at the designated target region in the brain.

## 2 Material and methods

### 2.1 Irradiation setup

**Figure 1:**
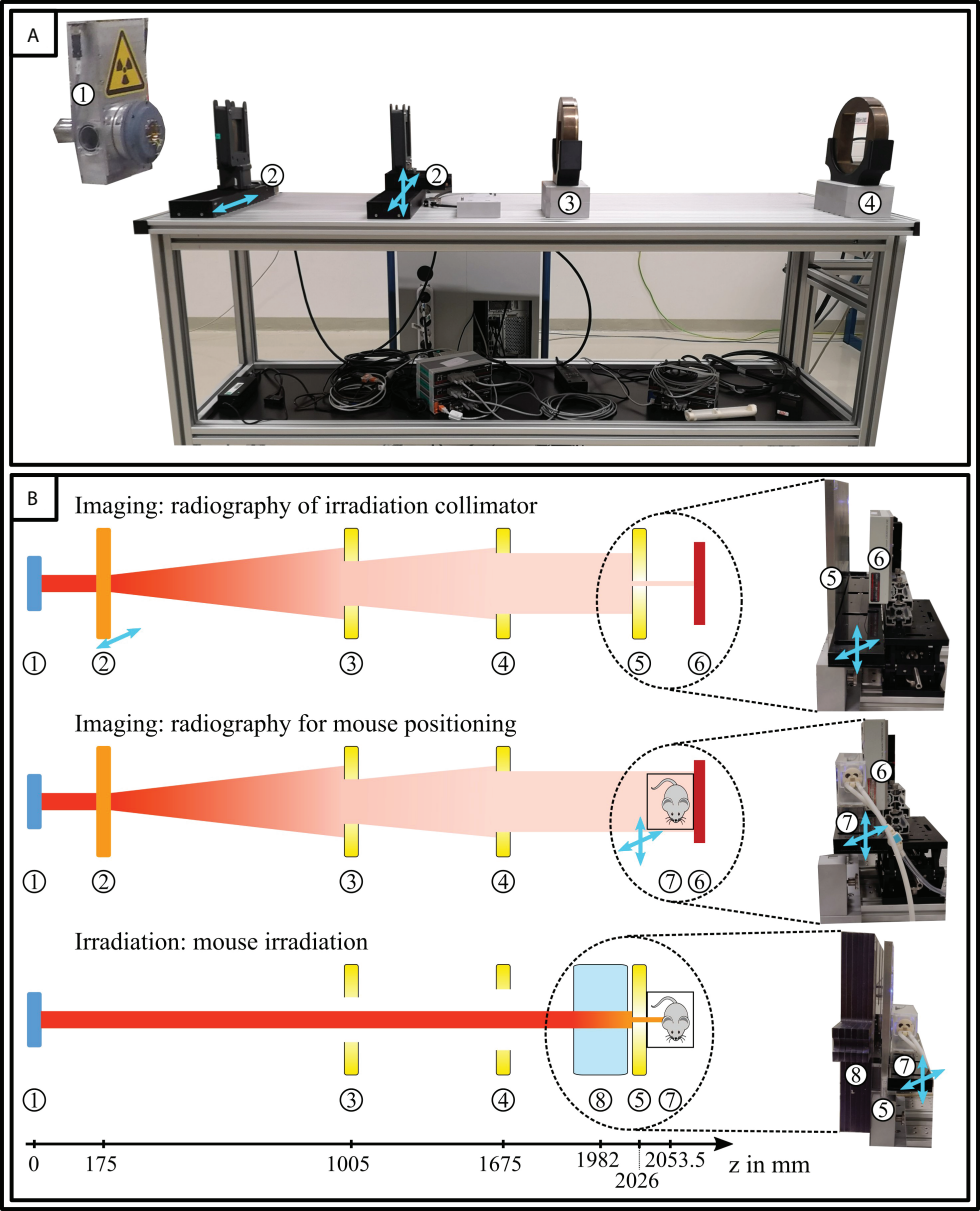
Irradiation setup for mouse irradiation. **(A)** Beamline of the irradiation setup with **(B)** schematic overview of possible modes of operation. The components are (1) the proton beam nozzle, (2) scatterers, (3) first (7.75×7.70 cm^2^) and (4) second collimator (11.50×11.55 cm^2)^, (5) irradiation collimator (Ø × 3.0 or 4.0 mm), (6) flatpanel detector, 7) animal stage with mouse in transport box, and (8) PC range shifter. The animal bedding unit and the beam scatterers are mounted on motorized stages for quick position adjustment and switching between modes of operation (“Imaging” and “Irradiation”), respectively. Only the first scatterer is needed for radiography.

Mouse irradiation was performed at the horizontal fixed beamline in the experimental hall of the Universitats Protonen Therapie Dresden (UPTD). The irradiation setup, its basic components, inline treatment planning, and positioning verification workflow have been described previously (16–18). The partly remodelled setup used for this manuscript consists of beam-shaping elements, an animal positioning stage, and a flat panel detector (see **Figure 1**). The scatterers are mounted on motorized stages for quick position adjustment. The detector is fixed on an optical table, can be moved manually and shielded from radiation when necessary. The setup allows for two principal modes of operation, i.e. “Imaging” and “Irradiation”.

The elemental compositions and dimensions of the beam-shaping elements, i.e., the scatterers and the collimators, have been previously described by Helmbrecht et al. (18). For mouse brain irradiation, an aluminium collimator (12mm thickness) with a circular aperture of either 3*mm* or 4*mm* diameter was added adjacent to the mouse bedding unit. To ensure the correct location of the Bragg peak within the mouse brain, polycarbonate (PC) pc plates (thickness: 46.51mm, water equivalent thickness: 53.16mm for 90MeV) acting as a range shifter were placed in front of the irradiation collimator. The appropriate thicknesses for the shifter have been determined as previously described (16) by characterizing the beam with a Giraffe multilayer ionization chamber (IBA Dosimetry, Schwarzenbruck, Germany) and EBT3 dosimetry films (Ashland Inc., Wilmington, Delaware, USA). For C57BL/6 mice, an additional 1.38mm pc slab was added in the beam path onto the transport box due to their smaller body size.

The two scatterers and the animal bedding unit are mounted on precision (<25µm) linear stages (LTM80F-300-HSM, LTM80F-100-HSM, LTM80P-75-HSM, OWIS GmbH, Staufen im Breisgau, Germany) for quick lateral displacement. The position of all axes can be changed remotely by the in-house developed interfaces *ScattERR* (https://github.com/schneidorlein/ScattERR) and *RadiAiDD* (https://github.com/jo-mueller/RadiAiDD, Version 0.1.0). The mouse was placed within a multi-modality bedding platform; the components and features as previously described by Müller et al. (19).

### 2.2 Proton radiography image acquisition

For on-beam radiographic imaging of mice, we deployed a C9320DK-02 CMOS flat panel detector (Hamamatsu Photonics K.K., Hamamatsu City, Japan) that features 10321012 pixels with a pitch of 0.05 mm to acquire images with a size of 52.8x52.8 mm^2^ and a frame rate of 8.4 Hz. The software for detector read-out was custom written in-house using the National Instruments Software Interface NI-IMAQ (version 3.7, National Instruments Corporation, Austin, USA).

To assess the image quality as a function of the beam parameters (i.e., beam energy and fluence), a MicroCT hole grid phantom (QRM GmbH, Mohrendorf, Germany, see **Figure 3C**) and a rectangular polymethylmethacrylat (PMMA) phantom (see **Figure S1**) were placed at irradiation position. Radiographic images were then acquired at proton energies of 150 MeV and 200 MeV to calculate the signal-to-noise ratio (SNR) and contrast-to-noise ratio (CNR) as follows:


(1) $$snr=\frac{S}{\sigma },\;cnr=\frac{S_{a}-S_{b}}{\sigma },$$

where S and σ refer to the mean signal value and the respective standard deviation. a and b denote the respective values from adjacent regions of high and low contrast.

The lead grid phantom L659036 (PTW Freiburg, Freiburg, Germany) features line pairs with a resolution of 0.6 mm^-1^ to 6.0 mm^-1^. Radiographic images were acquired to resolve the optimal distances between first scatterer, radiography object, and detector. The grid phantom was imaged at increasing distances (23 mm-100 mm) from the detector using a 200 MeV proton beam at 0.1 nA resulting in a dose of 19.8 mGy for an irradiation time of 10 s.

Moreover, the CT phantom was used to measure the visual resolution as a function of the applied dose. Here, the CT phantom was placed at a detector-object distance of 20mm, which corresponds to the animal’s position during irradiation. Dose deposition by radiographic imaging was determined with a PinPoint 3D ionization chamber (Type 31014, PTW Freiburg, Freiburg, Germany) at mouse position according to beam quality correction factor given in TRS-398 (20).

### 2.3 Animals

All animal experiments were approved by the local authorities (Landesdirektion Sachsen, DD24.1-5131/394/50 and DD24.1-5131/449/32) and conducted according to national (TierSchG) and European (EU Directive 2010/63/EU) animal welfare guidelines. Female C57BL/6JRj and C3H/NeNRj mice were supplied by Janvier Labs (Saint Berthevin Cedex, France) at least one week prior to the start of the experiments. The age at brain irradiation was 8-14 weeks. Animals were kept at a 12/12 h light/dark cycle in Euro Standard Type III cages with food and water *ad libitum*. Nesting material and igloos were provided as cage enrichment.

### 2.4 Irradiation workflow

The workflow for small animal irradiation consists of the consecutive steps of planning, positioning, and irradiation.

#### 2.4.1 Planning

**Figure 2:**
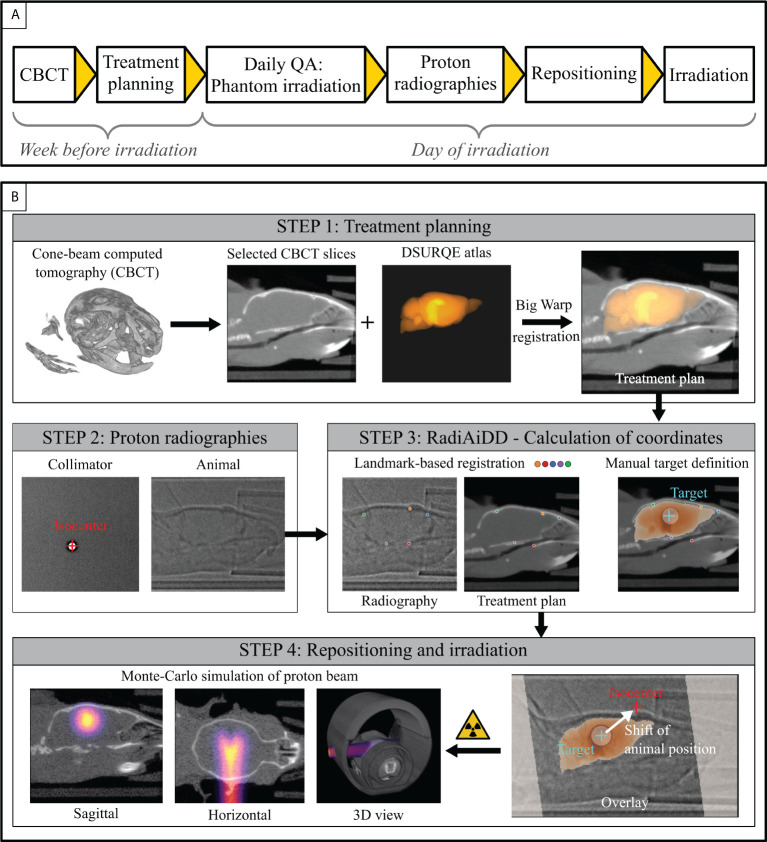
Workflow for proton brain irradiation. **(A)** Schematic diagram of the consecutive steps. **(B)** Individual steps for planning and repositioning. A CBCT serves as basis for treatment planning to the DSURQE brain atlas in the week prior to irradiation. Directly before irradiation, proton radiographies of collimator and animal are acquired to derive the beam isocenter and the animal repositioning parameters, respectively. Registration of the on-beam image with the treatment plan is performed based on five manually placed landmarks. The target coordinates are defined by positioning the collimator location onto the target area (i.e., the hippocampus). Then, the table coordinates for correct proton irradiation of the target area are calculated and the animal is shifted to this position. Post irradiation, Monte Carlo simulations compute the dose distribution within the brain based on the CBCT.

A cone beam computed tomography (CBCT) scan of each animal within the bedding unit was acquired one week before irradiation for determination of target position and later dose simulation using the small animal image-guided radiation therapy system (21). Imaging took place under isofluorane anesthesia (1.5-2% vol in O_2_) with the mouse in the multi-modality bedding unit (19). Subsequently, 15-25 sagittal slices from the reconstructed CBCT image data showing the central planes of the mouse skull, were averaged using a maximum intensity projection. Similarly, we created a multilabel image from the DSURQE anatomical brain atlas (22–26) (https://wiki.mouseimaging.ca/119 display/MICePub/Mouse+Brain+Atlases) and generated a sagittal maximum projection. The resulting two-dimensional label image features three labels, namely hippocampal region, rest of the brain, and background. The Big Warp (27) plug-in of Fiji (ImageJ Version 1.53d or higher, 64-bit Windows) (28) was used to register the two projected images, with the CBCT- derived image serving as fixed image. The resulting transformed image was then used as treatment plan image A. **Figure 2** gives an overview of the treatment planning pipeline and shows exemplary registered image data.

Inter- and intra-observer variation within this procedure were analyzed by a planning study on a subset of 10 animals. The animals were chosen so that both strains, different beam times, and different “difficulty levels” (i.e. how well the mouse was (re)positioned inside the bedding unit) were included. Three observers (MoS, TS, JM) of varying experience performed the planning procedure for each of the animals. In addition, each animal was planned three times by one observer on three separate days. Inter- and intra-observer agreement were quantified by calculating the Jaccard coefficients *J_i_* for each observer for the target region of interest, i.e., the projected hippocampal region. The majority-voted plan **F** for a particular animal served as the reference planning image:


(2) $$J_{i}=\frac{\left|A_{i}\cap F\right|}{\left|A_{i}\cup F\right|},\;F=majority(A_{1},A_{2},A_{3}),$$

#### 2.4.2 Positioning

Before positioning the animal at the beam site, a proton radiograph of the irradiation collimator (see **Figure 1**) was acquired to determine the treatment beam position. Then, the irradiation collimator was removed and the animal (within the bedding unit and transport box) was placed on the positioning stage for radiographic imaging. Subsequently, the *RadiAiDD* software was used to calculate the correct stage position for brain irradiation. For this purpose, the treatment plan image A was aligned with the acquired proton radiography of the mouse using a landmark-based similarity transformation (isocentric scaling, rotation and translation). The correct stage position for irradiation can then be calculated from the isocenter position obtained from the radiographic image of the irradiation collimator and the obtained transformation parameters.

Similar to the planning process, positioning involves manual steps that introduce inter- and intra-observer variations. To elucidate these variations, a registration study on the same subset of animals was conducted as described above. We then evaluated the variations in each of the manually set parameters, the calculated transformation parameters and the resulting motor stage coordinates.

#### 2.4.3 Irradiation

After moving the animal to the determined treatment position, irradiation was performed according to (16) with 90MeV protons. For this, the animals were anesthetized with Ketamine/Xylazine (i.p.; 100ml/10ml per kg body weight) and positioned into the multi-modality bedding unit and the transport box (19). Eyes were protected from drying out with Bepanthen eye ointment (Bayer Vital GmbH, Leverkusen, Germany). Anesthetized animals were ventilated with surgical air and heated during the entire transportation and irradiation procedure. The C3H/HeN mice used for histology in this paper were irradiated using a collimator of 3mm diameter at a dose rate of 3.3Gy with a single fraction of 8Gy. Animals were sacrificed by cervical dislocation 30min post irradiation.

#### 2.4.4 Daily QA

The positioning workflow was tested daily by irradiating a high-Z target (steel ball in the rectangular phantom, see **Figure S1**). We furthermore inserted EBT3 dosimetry films (Ashland Inc., Wilmington, Delaware, USA) into the phantom behind the steel ball. Correct planning and irradiation resulted in a black collimator-sized spot with the shadow of the steel ball visible as unirradiated area in the spot’s center (see **Figure S1**).

### 2.5 Monte Carlo simulation

Dose and (LET) simulations were performed using the (TOPAS) (29) software (version 3.6.p1) with default physics settings optimized for proton therapy (30). A previously validated dedicated beam model of the setup (16) was used to calculate dose and let distributions in the mouse CBCT image. Following the technique developed by Schneider et al. (31), the converter integrated in TOPAS was used to convert the CBCT data to stopping power ratio data. The positioning of the CBCT in TOPAS was based on the target coordinates determined by *RadiAiDD*.

### 2.6 Immunohistochemistry

Excised brains were fixed in 4% formalin for approximately 24h at room temperature and processed for paraffin-embedding in a semi-enclosed Benchtop Tissue Processor (Leica Biosystems, Wetzlar, Germany). Tissue sections of 3μm thickness were cut throughout the whole brain in either the sagittal or the horizontal direction with a distance of 100 µm (equates to approximately 150μm in fresh tissue) and mounted onto Starfrost Advanced Adhesive microscope slides (Engelbrecht GmbH, Edermünde, Germany, 11270). For immunohistochemistry, sections were dewaxed in xylene and rehydrated in a decreasing ethanol series and pbs. Heat-induced antigen retrieval was performed by boiling in citrate buffer (pH 6) for 20min. Sections within the buffer were allowed to cool on ice for at least 15min and then washed in pbs. Subsequently, 1x Rotiblock (Carl Roth, Karlsruhe, Germany, A151) supplemented with 0.1% Triton X-100 (SERVA Electrophoresis GmbH, Heidelberg, Germany, 37240) was applied for1h. Antibody incubation was conducted in 1x Rotiblock at room temperature for 1h each; using rabbit anti-*γ*H2AX antibody (Bethyl Laboratories, Montgomery, USA, IHC-00059, dilution 1:500) as primary and anti-rabbit AlexaFluor488 (Thermo Fisher Scientific, A11034, dilution 1:500) as secondary antibody. Sections were rinsed with pbs in between all staining steps. Counter-staining in 4’,6-diamidino-2-phenylindole (DAPI) was applied for 10 min before coverslipping with fluorescence mounting medium (Agilent Technologies, Santa Clara, USA, S302380).

### 2.7 Microscopy image acquisition and analysis

Image acquisition was performed with a 20x objective (Plan-Apochromat 20x/0.8 M27, Carl Zeiss AG, Oberkochen, Germany) at the Axio Scan.Z1 digital slide scanner (Carl Zeiss AG, Oberkochen, Germany) by the Light Microscopy Facility of the Center for Molecular and Cellular Bioengineering (CMCB). The used software was Zeiss ZEN 3.1 (blue). Excitation/emission wavelengths were 353nm/465nm and 493nm/517nm for DAPI and AlexaFluor488, respectively. Images were stored with 16-bit depth and a pixel size of 0.325μm x 0.325μm using an ORCA-Flash 4.0 V3 Digital CMOS camera (Hamamatsu Photonics K.K., Hamamatsu City, Japan). For microscopy image representation, the data was post-processed (i.e., brightness-contrast adjustment and background removal) using Zeiss ZEN 2.3 lite and Fiji ImageJ (version v1.52n). Images were analyzed with the pipeline described in (16). In brief, the spatial relative DNA damage distribution was calculated (https://github.com/Theresa-S/Cell-ratio-detection) using optimized prominence values for the Maxima Finder (DAPI: 600; *γ*H2AX: 1400; see **Figure S2**) and a tile size of 256 x 256 pixels. The relative DNA damage maps were subsequently registered to the CBCT of the respective animal using the Slice2Volume workflow (https://github.com/jo-mueller/Slice2Volume). This enables the overlay of the induced relative DNA damage to the applied dose for a pixel-wise correlation.

## 3 Results

### 3.1 Optimized setup and imaging workflow

#### 3.1.1 Setup adaptation

**Figure 3:**
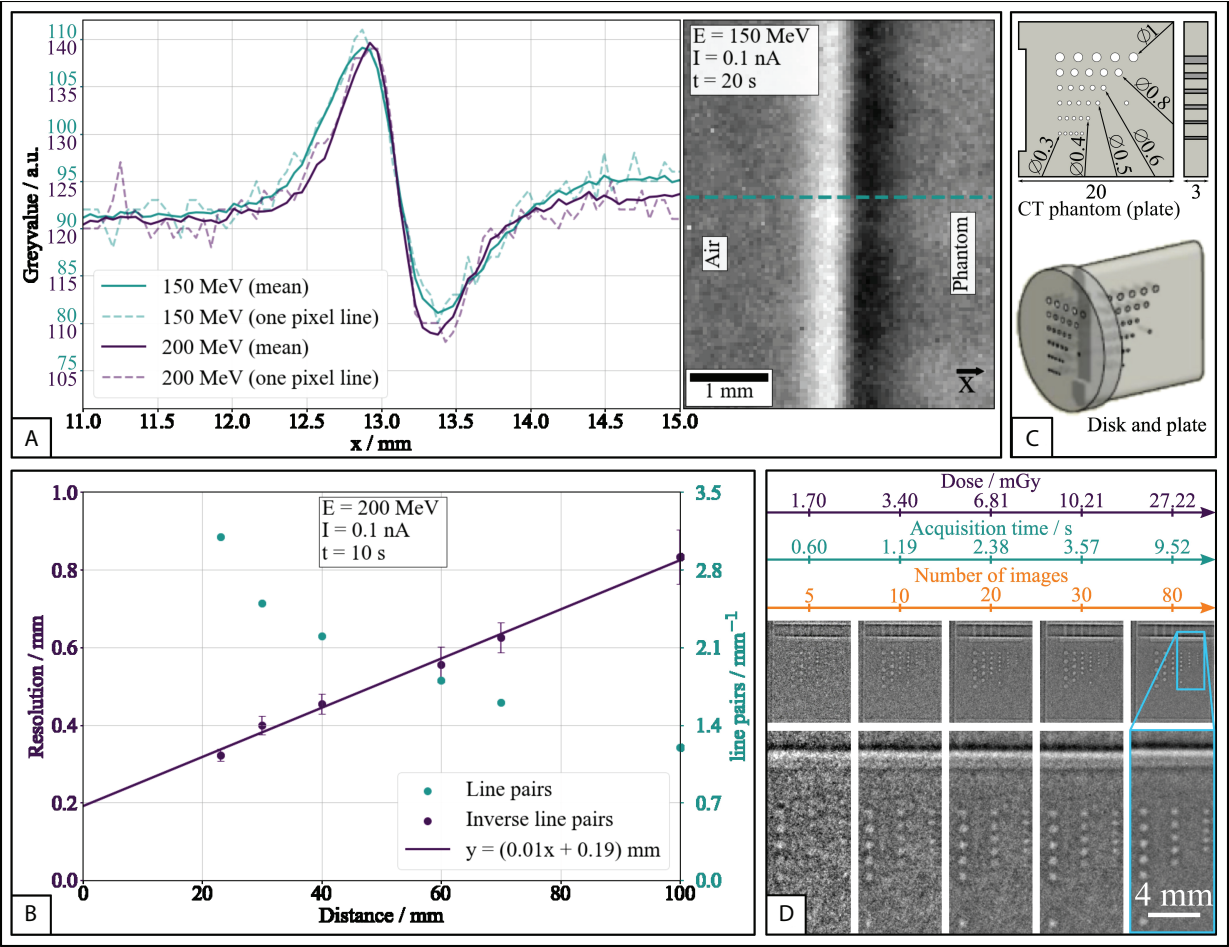
Results of proton radiography resolution experiments. **(A)** Proton fluence at the edge between air and phantom (PMMA) for two beam energies either along one pixel line (dashed) or as mean over 100 pixel lines (solid). **(B)** Image resolution and line pairs determined from the lead grid phantom as a function of distance between phantom and flat panel detector. **(C)** Schematics of the used MicroCT hole grid phantom. The hole pattern in the disk is analogous to the plate. All units are given in mm. **(D)** Image resolution determined from the hole phantom as a function of applied dose, acquisition time, and number of acquired frames.

**Figure 3A** shows the border between the CT phantom and air at 150MeV and 200MeV. The scattering in the phantom causes an inhomogeneous proton fluence at the phantom’s edge with a loss of fluence in regions of dense materials (PMMA) and an increase of fluence in the surrounding regions. The comparison of both energies reveals that the peak-to-valley edge width increases at lower energy (peak difference ΔFWHM = 0.08mm between the two energies), which is detrimental to the resolution and visibility of more detailed structures.

The resolution increases for smaller distances between object and detector (see **Figure 3B**). The smallest possible distance between object and detector that satisfied the spatial constraints given by the experimental setup (mainly the transport box) was 23mm. To minimize scattering, the thickness of the transport box wall adjacent to the radiography detector was reduced to 1mm.

The investigation of SNR and CNR shows that both depend on dose and proton energy for acquisition times up to 19s. SNR and CNR at 200MeV are larger than at 150MeV (see **Figure S3**). The detectability of details in the hole grid phantom (see **Figure 3C**) increases with increasing number of frames (and, hence, acquisition time) and dose (see **Figure 3D**), due to the decreasing noise and thereby increasing SNR and CNR. The smallest apertures of 0.3mm diameter are clearly recognisable and distinguishable from the noise after approximately 30 frames, 3.6s, and 10.2mGy. Since image quality correlates with the applied dose (see **Figure 3D**), a proton energy of 200MeV, beam current of 0.1nA, and acquisition time of 8s were chosen for animal radiography to limit the radiation exposure to approximately 23mGy while accomplishing a high quality radiography.

#### 3.1.2 Imaging workflow

After adjusting the physical parameters for animal proton radiography, the imaging process was optimized. Obtaining a homogeneous background intensity in the acquired images required to subtract both the off- and on-beam image background. To correct for the detector-induced background signal (*I*_*dark*_ ), 300 frames were recorded and averaged prior to proton imaging. Next, the beam fluence-induced image background *I*_*beam*_ was determined by averaging another set of 300 frames at (200MeV, 0.1nA, 30s). Both background images *I*_*dark*_ and *I*_*beam*_ are automatically subtracted from all subsequently acquired frames. The obtained background-corrected frames can then be combined by calculating the median pixel values acrossall frames for robustness against salt-and-pepper noise.

#### 3.1.3 Planning study and beam target variability

**Figure 4:**
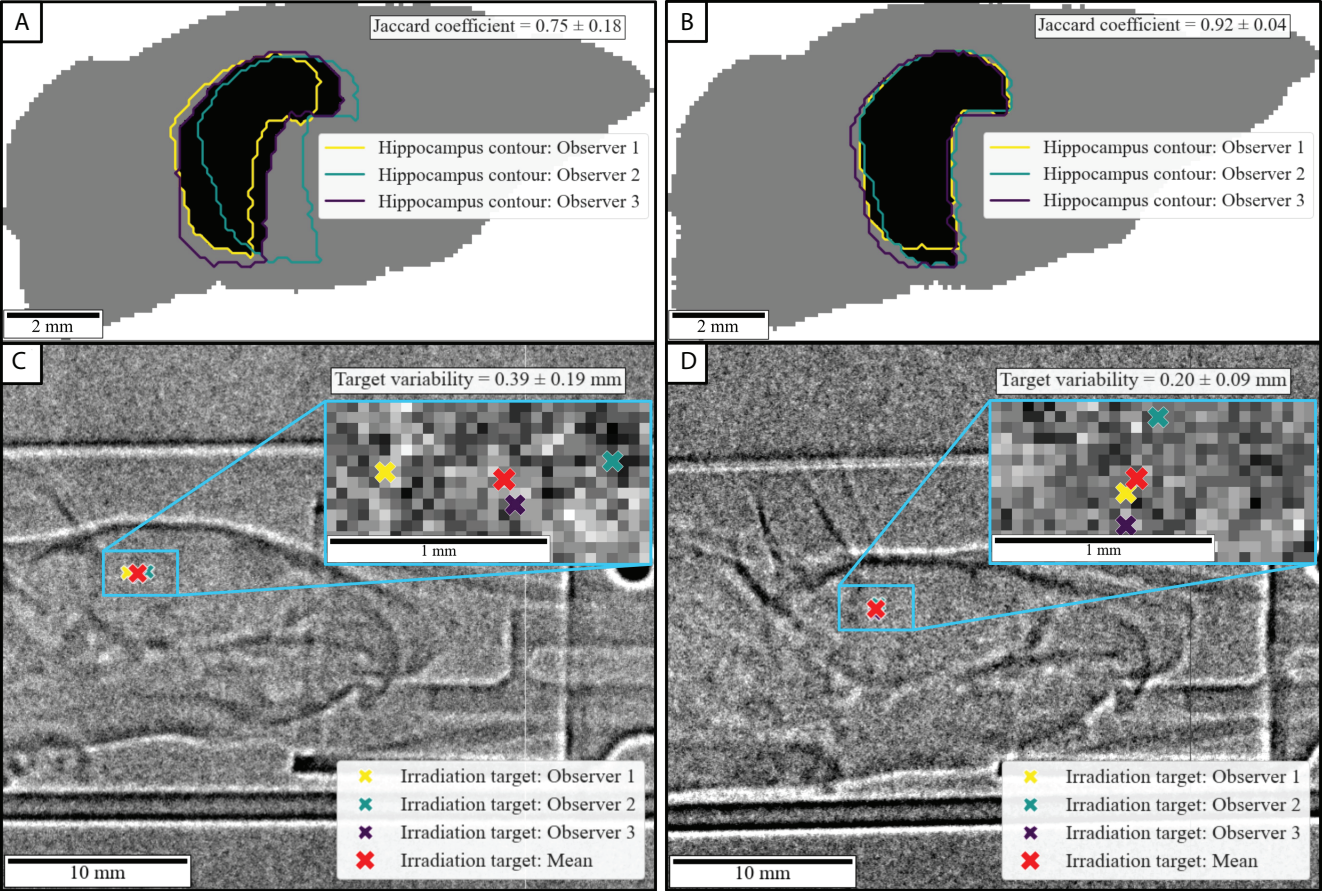
Representative variability in planning and registration. The upper row shows inter-observer variations and Jaccard coefficients of the projected target region for the animals with **(A)** the lowest and **(B)** the highest inter-observer agreement. In the lower row, **(C)** and **(D)** show the resulting variation of the registration-derived target coordinate for the animals in **(A)** and **(B)**, respectively.

The Jaccard coefficient for the inter- and intra-observer variability in the planning process was 0.84±0.10 and 0.92±0.05, respectively. To visualize the extent of the occurring deviations, the projected plan datasets resulting in the lowest and highest Jaccard coefficients are shown in **Figure 4A, B**. The animal-wise Jaccard coefficients for inter- and intra-observer variations are shown in the **Supplementary Materials** (**Figure S4**).

We furthermore determined the inter- and intra-observer variations of the target coordinates, which were calculated during the repositioning process. The inter- and intra-observer variabilities were 0.26±0.10mm and 0.22±0.10mm, respectively. The resulting irradiation target coordinates obtained by all observers overlaid with the respective radiographic images are shown in **Figure 4C, D**) for the same selected mice. The animal-wise inter- and intra-observer variations for the calculated target coordinates as well as all derived transformation parameters are depicted in the **supplementary materials** (**Figure S5-S11**).

### 3.2 Biological verification

**Figure 5:**
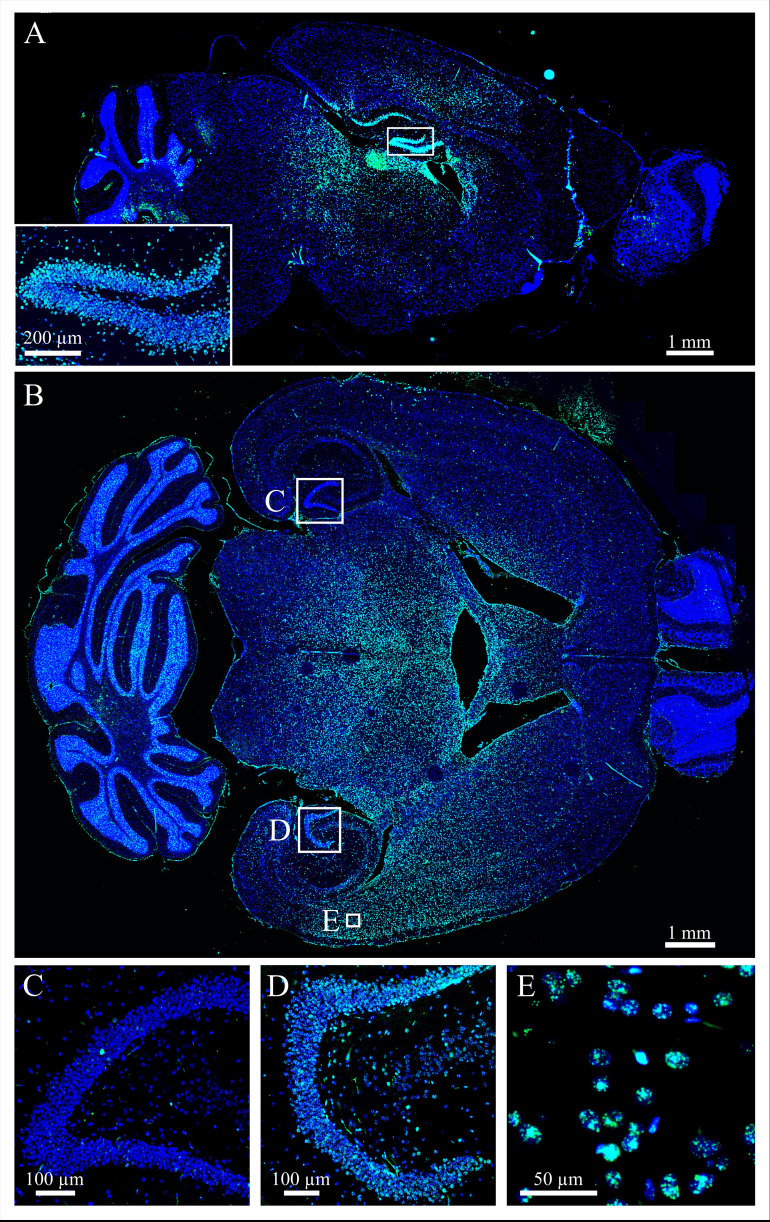
Biological verification of the irradiation workflow. DNA damage in the brain cells (DAPI, blue) was stained *via* γH2AX (green) 30 min after 8 Gy irradiation. **(A)** The sagittal section shows that the beam was correctly applied to the target location, the hippocampal area. **(B)** The horizontal section additionally reveals that the proton beam stops in the middle of the brain. The **(C)** non-irradiated hippocampus has no elevated damage, whereas the **(D)** irradiated one shows increased *γ*H2AX expression. **(E)** Higher magnification of cells in the beam path visualize radiation-induced foci.

The correct beam application was verified biologically in two irradiated brains. Sagittal (see **Figure 5A**) and horizontal (see **Figure 5B**) slices of mouse brains visualize the position of the proton beam in x, y (anterior-posterior, dorsal-ventral) and x, z (anterior-posterior, depth) directions, respectively. The distribution of radiation-induced DNA damage (see **Figure 5**) proves that the animals were hit in the hippocampal target location and that the beam stops within the brain.

**Figure 6:**
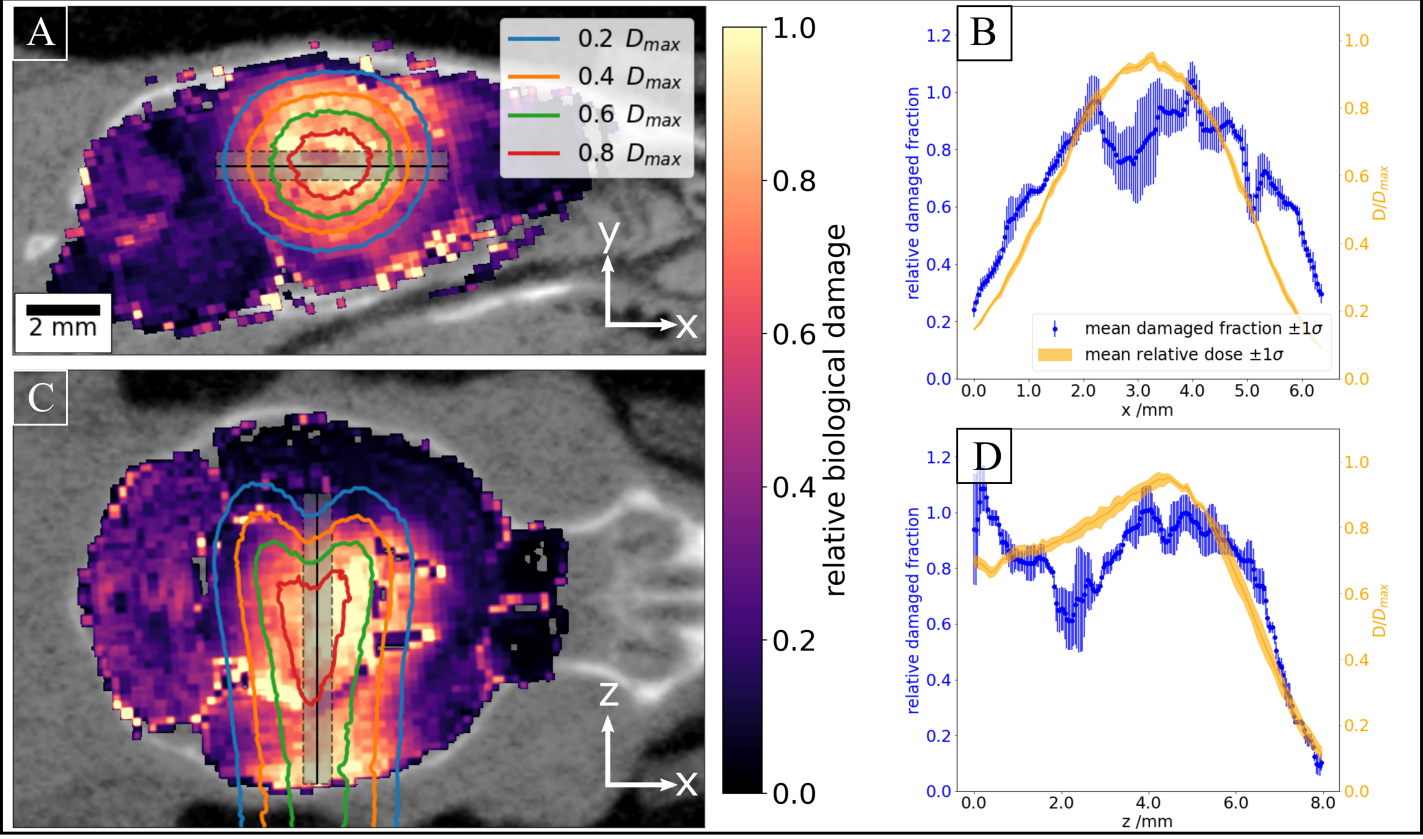
Correlation of biological damage with applied dose. **(A)** Overlay of aligned relative DNA damage and simulated dose in a representative sagittal plane. The grey box indicates the profile line along which average dose and relative biological damage have been calculated as shown in **(B)**. **(C)** and **(D)** show the respective plots for a coronal plane.

We then evaluated the relation between applied dose and biological effect. **Figure 6** shows the resulting overlays of relative biological damage and simulated dose for two representative sections in the sagittal and coronal plane, where the cell distribution enabled direct analysis. Image analysis of DNA damage using the described analysis workflow clearly visualizes the beam path in the tissue. The underlying cellular composition of the brain tissue introduces variation in the biological read-out, which impacts the results to varying degrees depending on the analyzed brain section (data not shown). The profile lines, however, indicate a good correlation between deposited physical dose and induced biological damage.

## 4 Discussion

Research questions in proton radiooncology aim to address important aspects of clinical outcome and require preclinical *in vivo* data. These questions include the RBE of protons (32), proton-specific normal tissue damage, and novel combination treatments (33). One challenge for such experiments is the precise irradiation of small animal models, which is required for meaningful translation into clinical trials due to the necessary down-scaling of spatial dimensions. The goal of the presented study was to develop an improved positioning workflow by including on-beam proton radiography of living mice into the existing pipeline (16, 17). The achieved increase in reproducibility and precision, as well as the streamlined irradiation workflow will enable further preclinical studies requiring high precision proton irradiation of small animals.

The presented irradiation setup puts high value on cost-effectiveness, relative technical simplicity, and open code distribution to facilitate reproduction and adaptation. The used components are readily-available to allow implementation with little technical preconditions at other experimental centers with quasi mono-energetic proton beams. In addition, the redesigned radiation setup provides novel opportunities to external users of our facility, e.g. through European transnational access of the INSPIRE network (34). The applied software for planning (Big Warp, Fiji (27, 28)), registration (*RadiAiDD*), and component interaction (*ScattERR*) are open source. The operation modes of the setup (“Imaging” and “Irradiation”) allow for image acquisition of setup and animals, repositioning, and irradiation. The usage of motorized elements enables remote control of the used components and thereby ensures radiation protection of the personnel. Quality assurance of the workflow, similar to the clinics (35, 36), is performed daily with phantom irradiation.

Benchmark experiments were performed to determine the optimal radiography acquisition parameters for beam energy, dose, and object-detector distance. As a result of these experiments, we provide setup parameters that allow for sufficiently high-resolution radiographic imaging with a commercial flat panel detector, while exposing the targets to reasonable (low-LET) proton radiation doses of approximately 23mGy. If necessary, this dose can be further reduced by shielding of the animal body outside the field-of-interest. The chosen beam energy of 200MeV can be delivered by common clinical and experimental cyclotrons. The achieved spatial resolution of 0.3mm allows to resolve relevant anatomical landmarks. In comparison to other reported ion-based radio- or tomographic techniques (14, 37–39), our method yields competitive resolution. The implemented method benefits considerably from placing the detector close to the object and exploiting changes in proton fluence rather than particle stopping, which is consistent with the theoretical description (40) as well as previously reported experiments regarding object-detector distance (17). Due to the requirements regarding object-detector distance and object thickness, the used radiographic method may not be viable for clinical use. Preclinical experiments, however, provide suitable physical and spatial conditions to exploit the used mode of proton radiographic images for high-precision positioning and irradiation. The choice of beam energy and acquisition time had to be weighted against the applied dose. Using the plateau-region of the beam in a shoot-through fashion allows to achieve the above-reported resolution at a dose of approximately 23mGy. This represents an improvement over other reported implementations (e.g., Darne et al. (38): 47.2 mGy, Harms et al. (41): 50mGy). Technically highly sophisticated techniques yield low per-image doses at high beam energies (Durante et al. (13), 10mGy, 800MeV), but do not provide the cost-effectiveness or availability, which are key features of the presented method.

The planning study shows inter- and intra-observer variations in the registration of brain atlas and CBCT, which impact the resulting overall targeting accuracy. In general, the inter-observer variability was lower than the intra-observer variability (mean Jaccard indices: 0.84±0.10 vs. 0.92±0.05), which highlights the highly subjective perception of sufficiently good agreement when performing registration tasks. Since the true registration is not known in such scenarios, this is an inherent problem of both manual and automated registration (42) and subsequently applies to the performed planning experiment. The inter- and intra-observer Jaccard coefficients obtained in this study are comparable to similar clinical investigations. Wohlfahrt et al. (43) found a Jaccard coefficient of 0.80±0.05 for an experienced radiooncologist contouring the tumor gross volume of lung cancer on two different days. For 3D-CT contouring of pancreatic ductal adenocarcinoma, Choi et al. (44) noted inter-observer Jaccard coefficients of 0.521-0.783 for three medical specialists. Alasti et al. (45) reported Jaccard coefficients of 0.665-0.811 between different observers and 0.851-0.917 for repeated delineations by the same observer for CT contouring of prostate tumors. To address the variations in the present planning study, it is notable that the disagreement between observers occurs predominantly in the basal part of the hippocampus. In our case, the defined target region was located in the upper part of the hippocampus. Thus, the practically achieved planning precision may be higher as suggested by the calculated Jaccard coefficients. The registration and, consequently, the positioning procedure yielded good agreement between observers. The determined variations in the calculated beam target coordinates are consistently in the sub-millimeter domain (<0.41±0.19mm; mean inter- and intra-observer variations of 0.26±0.10mm and 0.22±0.10mm). While setups with on-site X-ray imaging provide slightly higher positioning accuracy [0.08mm (46) or 0.24mm (11)], such performances often come at the price of significant technical expense. It should be noted that the registration and positioning procedure encompasses the manual, interactive placement of the desired beam target location (with respect to the desired projected target region, i.e., the hippocampal region). Thus, the ensuing variability of the calculated target position is composed of variation of the derived transformation parameters as well as the chosen beam target. The implemented workflow allows for further simplifications that address this shortcoming to a certain degree. For instance, the target could be set automatically based on objective criteria such as the center of gravity of the hippocampal area. To further minimize intra- and inter-observer variability, observer training sessions prior to experimental campaigns are advisable (47).

It is worth mentioning, that – while allowing for high lateral precision in irradiation – the implemented radiographic method does not yield additional information for choosing the beam range. This is of particular importance for not well-localized targets such as orthotopic tumors in the brain, which can show considerable variation in their location and thus require an individualized irradiation field. While the measurement of water-equivalent thickness by means of proton radiography has been described previously (17), it is desirable to include tomographic information for the determination of the appropriate beam range. The irradiation of specific normal tissue regions in the brain as demonstrated in this manuscript, however, provides the necessary conditions for using a generalized beam range, which is highlighted by the overlay of simulated dose and DNA damage.

The DNA damage analysis was performed to verify our workflow using a pre-defined beam-range. Histology proved that the DNA damage induction was located in the delineated target region. After subsequent image analysis, a clear accordance was shown between the proton beam and radiation-induced DNA damage. Thus, we could replicate the data from our previous study on small animal irradiation (16) while streamlining the radiation workflow considerably. It has to be noted that despite parameter optimization, the algorithm used for calculating the relative DNA damage distributions has some weaknesses. The maxima finder, which is the core method for cell counting, does not perform equally well in regions of high or low cell density, or when inhomogeneous fluorescence intensity is present. Specifically, a slight overestimation of DNA damage was noted in unirradiated or low-dose irradiated brain areas. Nevertheless, the algorithm provides fast and objective analysis of spatial tissue characteristics, which we considered sufficiently accurate for the research question at hand, i.e., visualizing the proton beam *via* the induced DNA damage in the tissue. Relating the damage to the applied dose further revealed the translational value of our model: the congruence of physical effect and biological outcome is a valuable tool in elucidating underlying radiobiological mechanisms. Future studies could, for example, include the let or beam quality (48) to investigate the relation between the different physical parameters and the ensuing biological effects in a preclinical *in vivo* setting. One drawback of RBE studies in brain is the inherent heterogeneity of the organ. Differing cell compositions and densities, as well as the four brain ventricles complicate a clean correlation of dose and damage. Hence, irradiation of larger organs with high cellular homogeneity such as the liver could provide additional benefits for *in vivo* RBE investigations. On the other hand, moving organs are more difficult to irradiate and cannot be distinguished as clearly as the skull bones in proton radiography. Additionally, cranial tumors are often considered an indication for proton therapy; therefore, normal tissue studies on brain are highly needed. The optimal solution to handle organ heterogeneity is cell-wise dose mapping, where each cell is analyzed individually. This approach requires not only precise dosimetric measurements and dose simulations, but also high precision irradiation, which can be provided by our workflow.

In conclusion, we could show that high-precision subvolume irradiation of small animals can be achieved with image-guidance from proton radiography using a flat panel detector. The imaging dose was limited to an acceptable amount and biological validation proved successful treatment of the hippocampal target area. The robustness and cost-effectiveness of the setup and the streamlined and clinic-orientated workflow enable a wide range of preclinical proton experiments. Possible future applications include irradiation of larger animal cohorts, fractionated proton irradiation, additional normal tissue studies on other organs and sub-volumes, and the treatment of orthotopic tumors. This will facilitate animal studies in proton radiooncology and help to provide much needed *in vivo* data for a range of clinically relevant research topics.

## Data availability statement

The datasets presented in this study can be found in the online repository Zenodo under the access link doi: doi.org.10.5281/zenodo.6778020.

## Ethics statement

The animal study was reviewed and approved by Landesdirektion Sachsen.

## Author contributions

EBo, TS, EBe, AD, AL, and JM contributed to the conception and design of the study. TS, EBe, AD, and CN were responsible for animal ethics. EBo, EBe, MK, AL, and JM offered supervision during the project. Methodology was done by MoS, EBo, TS, JB, MiS, AL, and JM. Software was provided by MoS, JB, FT, and JM. MoS, EBo, TS, EBe, JB, AD, SG, LH, SN, and JM acquired data and MoS, TS, and JM were involved in data curation. Data analysis and interpretation were performed by MoS, TS, JB, and JM. MoS, EBo, TS, JB, and JM visualized the data and MoS, TS, LH, and JM wrote the original draft of the manuscript, EBe and AL contributed major revisions. All authors read and approved the final version of the manuscript.

## Funding

The experimental part of the University Proton Therapy Dresden (UPTD) facility has received funding from the European Union’s Horizon 2020 research and innovation program under grant agreement No. 730983 (INSPIRE).

## Acknowledgments

The authors would like to thank Dorothee Pfitzmann, Liane Stolz-Kieslich, Katja Schumann, and Daniela Pollack for their excellent technical assistance on animal experiments and histology. We would like to acknowledge the help of Anne Dreyer, Gerd Rothe and Michael Reiche from the mechanical workshop. This work was supported by the Light Microscopy Facility, a Core Facility of the cmcb Technology Platform at TU Dresden, especially by Ellen Geibelt. We further want to thank the Teams of University Proton Therapy Dresden (UPTD) and IBA for their support during the proton irradiation experiments.

## Conflict of interest

In the past 5 years, MK received funding for her research projects by Merck KGaA (2014-2018 for preclinical study; 2018-2020 for clinical study) and Medipan GmbH (2014-2018). She is involved in an ongoing publicly funded (German Federal Ministry of Education and Research) project with the companies Medipan, Attomol GmbH, GA Generic Assays GmbH, Gesellschaft fur medizinische und wissenschaftliche genetische Analysen, Lipotype GmbH, and PolyAn GmbH. For the present study, MK confirms that none of the above mentioned funding sources were involved.

The remaining authors declare that the research was conducted in the absence of any commercial or financial relationships that could be construed as a potential conflict of interest.

## Publisher’s note

All claims expressed in this article are solely those of the authors and do not necessarily represent those of their affiliated organizations, or those of the publisher, the editors and the reviewers. Any product that may be evaluated in this article, or claim that may be made by its manufacturer, is not guaranteed or endorsed by the publisher.
