# Supplementary material for: Combined proton radiography and irradiation for high-precision preclinical studies in small animals
Source: Front Oncol. 2022 Aug 31;12:982417. doi: 10.3389/fonc.2022.982417 (PMC9677333; doi:10.3389/fonc.2022.982417)
Supplement: Supplementary file 1 [file DataSheet_1.pdf]

Supplementary Material

1 PROTON RADIOGRAPHY IMAGE ACQUISITION

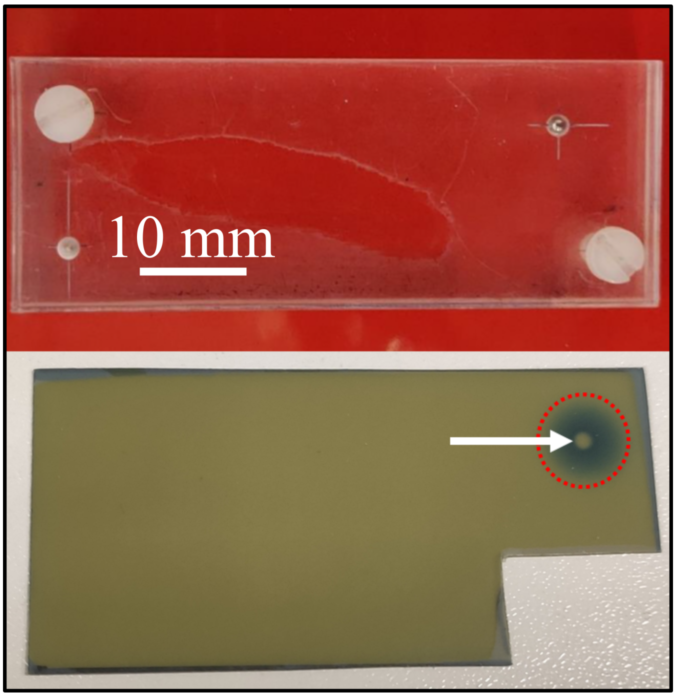

Figure S1: Phantom used for the daily quality assurance (QA) routine. An EBT3 radiochromic film inserted in the rectangular phantom containing a steel ball is used to validate the correct positioning workflow. When planning a treatment with the steel ball as target, correct irradiation yields a black circle (i.e. dose darkening the EBT3 film; dashed red circle) with an undarkened spot in the center (i.e. dose spared by the steel ball; white arrow).

2 MICROSCOPY IMAGE ACQUISITION AND ANALYSIS

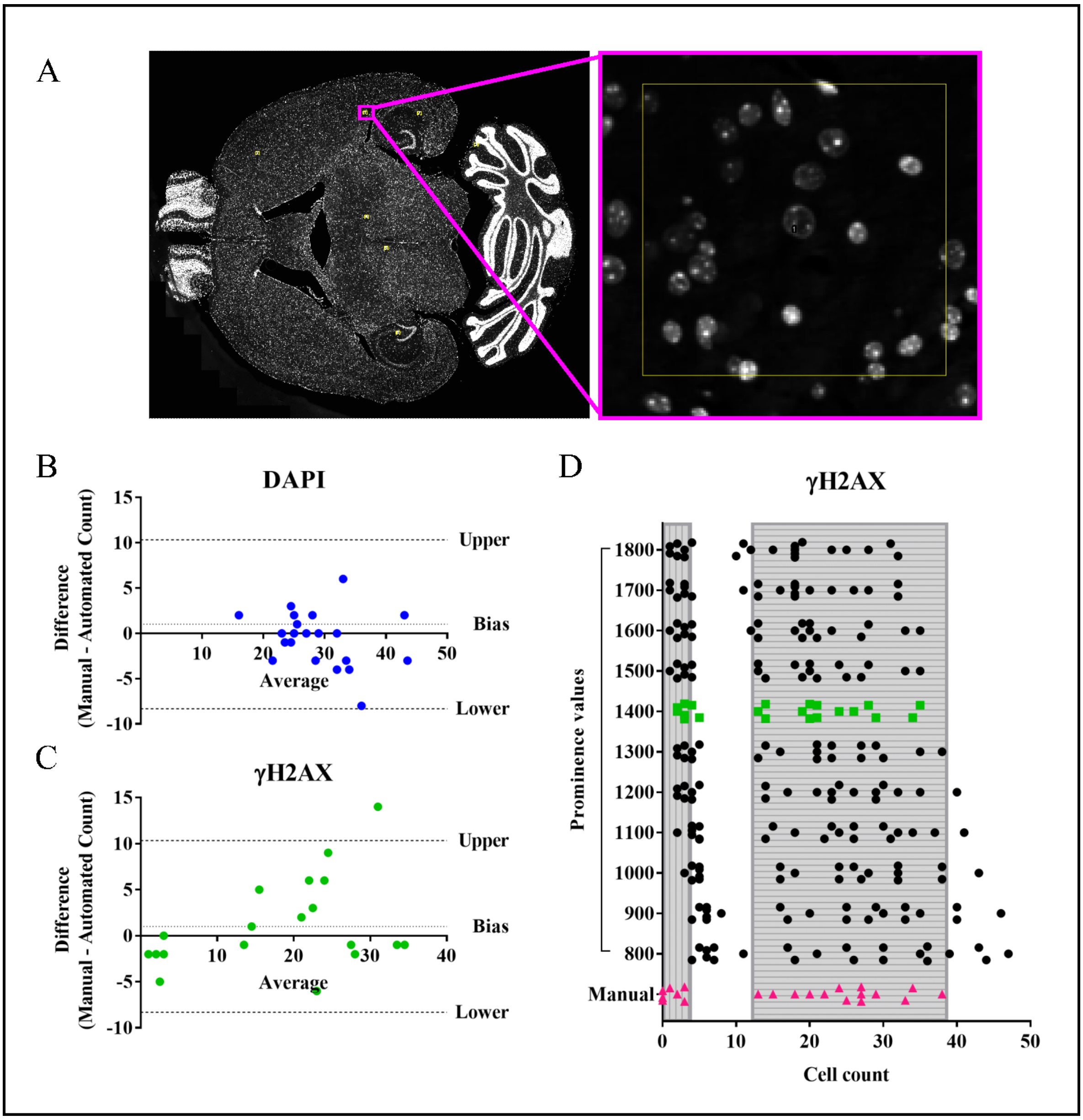

Figure S2: Parameter optimization for the DNA damage evaluation. (A) Exemplary region of interest in the DAPI channel. Seven regions of interest of approximately 40 x 40 pixels were counted per image and channel in three representative histological sections. The regions were located in brain areas of differing cell or DNA damage density. Bland-Altman plots of (B) DAPI and (C) γH2AX for the optimized prominence values 600 and 1400, respectively, show the difference between manual and automated counting. For the used parameters, the bias is close to zero and the 95% upper and lower limits of agreement are within acceptable range. (D) Results of γH2AX evaluation for varying prominence values (black dots, green rectangles) and manual counting (pink triangles). When analyzing cells with DNA damage, the algorithm tends to overestimate the numbers in regions of low density (i.e., the non-irradiated brain areas, vertical grey stripes) or underestimate the numbers in regions of high DNA damage density (grey horizontal stripes). A prominence value of 1400 yielded a good compromise between both (green rectangles).

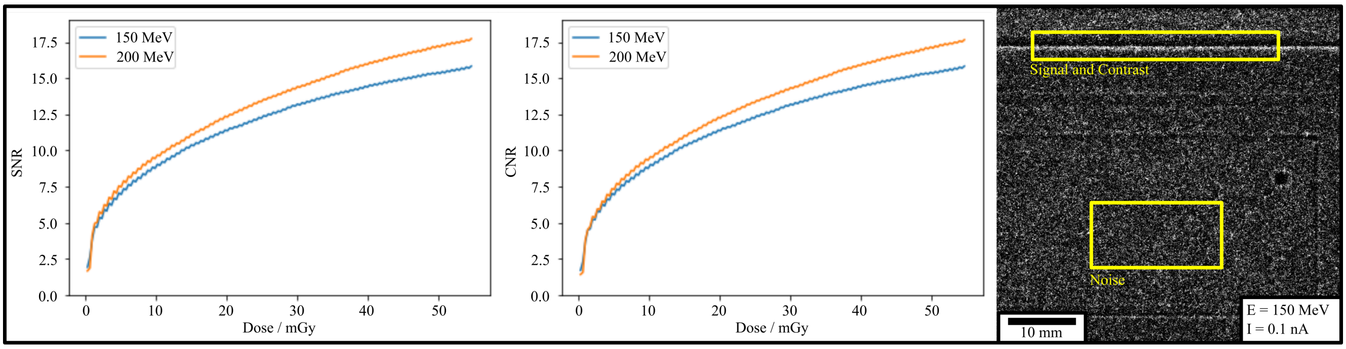

Figure S3: Signal-to-noise ratio (SNR) and contrast-to-noise ratio (CNR) dependence on dose at mouse position in radiographic imaging for two proton energies. One radiographic slice is shown on the right with the corresponding areas for SNR and CNR calculation.

3 IRRADIATION WORKFLOW

3.1 Planning

As described in the main manuscript, planning images were created by registering the projected atlas image data with the projected CBCT image data in the sagittal plane. This was repeated with three observers and we evaluated the planning variations in terms of Jaccard coefficients. For this, a reference planning image was calculated for every animal using a pixel-wise majority vote criterion. Both inter- and intra-observer variations were evaluated (see Figure S4).

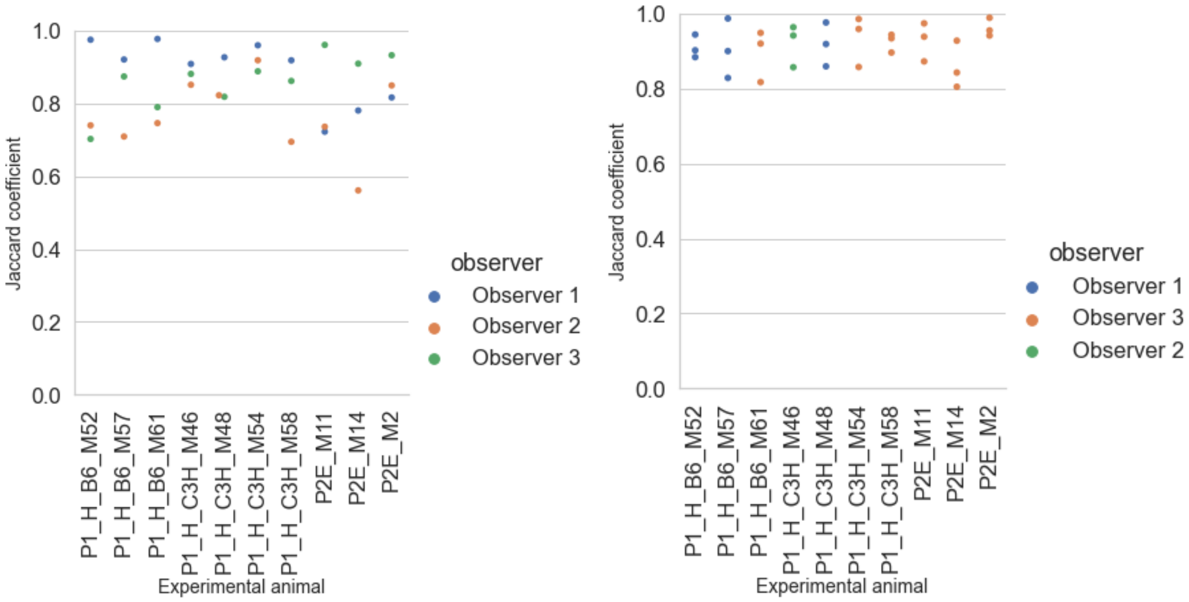

(a) (b)

Figure S4: Inter- (a) and intra-observer (b) variations between each plan and the majority-voted reference planning image.

3.2 Repositioning

We evaluated the positioning deviations resulting from inter- and intra-observer variations when registering the plan image data (i.e., the overlay of sagittal CBCT image planes and the projected atlas data) with the acquired proton radiography. The final target coordinates $\vec{M}$ that are passed to the motorized stage are calculated from the user-determined target coordinates $\vec{XR}$ with a similarity transform as follows:

$$\vec{RG} = s \cdot \mathbf{R}(\alpha) \cdot \vec{XR} + \vec{t} \tag{S1}$$

where s, **R**, α and $\vec{t}$ denote the scaling, rotation matrix, rotation angle and translation of the similarity transform. The rotation matrix **R** can be calculated from the angle α according to the following equation:

$$R(\alpha) = \begin{pmatrix} \cos(\alpha) & \sin(\alpha) \\ -\sin(\alpha) & \cos(\alpha) \end{pmatrix} \tag{S2}$$

The vectors in this equation have an x- and y- component, which refer to the horizontal and vertical axes in beam's eye view. Each component of the transformation (scaling, rotation and translation) features an observer-induced variability, the superposition of which compose the variation of the resulting target coordinates $\vec{RG}$. In this section, we show the component-wise inter- and intra-observer variations for each relevant parameter in both x- and y-direction.

3.2.1 Inter-observer variations

This section shows inter-observer variations for the above listed parameters $\vec{XR}$, s, α, $\vec{t}$ and $\vec{RG}$ separately in x and y, where applicable. The x- and y-coordinates refer to the horizontal and vertical axes in beam's eye view. For better visual comparison, the results were normalized to the animal-wise mean value for $\vec{XR}$, α, $\vec{t}$, and $\vec{RG}$.

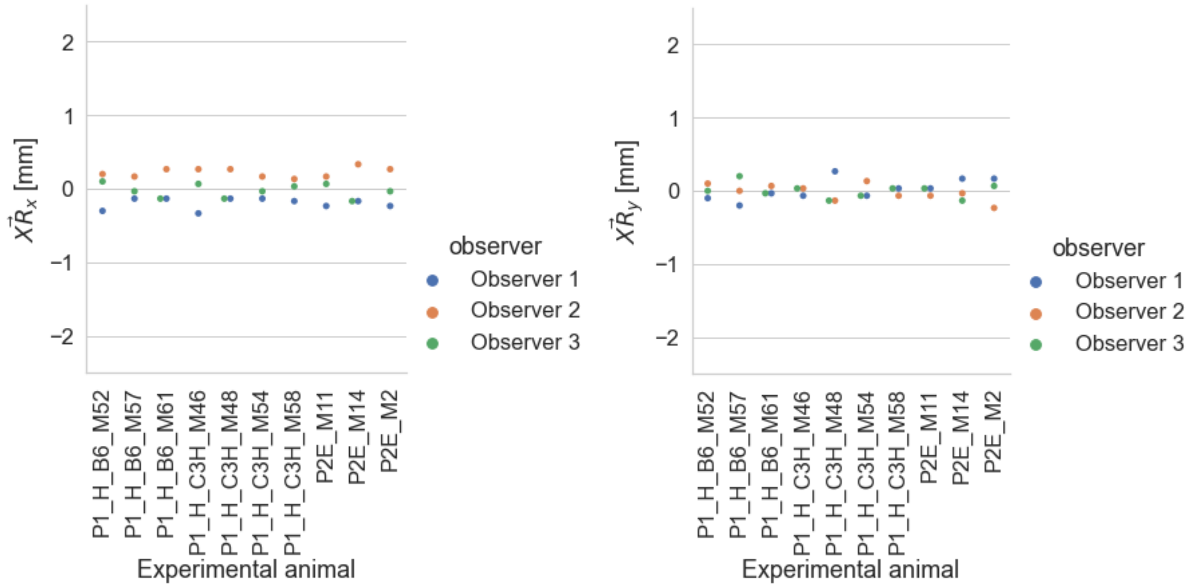

(a) (b)

Figure S5: User-determined coordinates $\vec{XR}$ in x (a) and y (b) in the CT-based coordinate system.

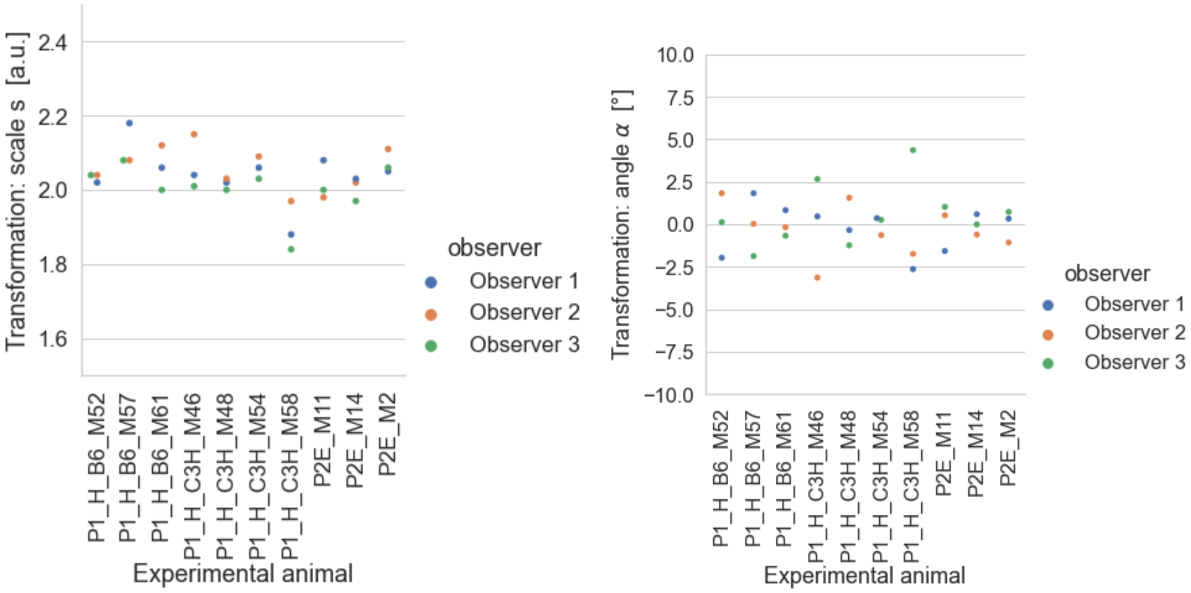

(a) (b)

Figure S6: User-determined scale s (a) and rotation angle α (b).

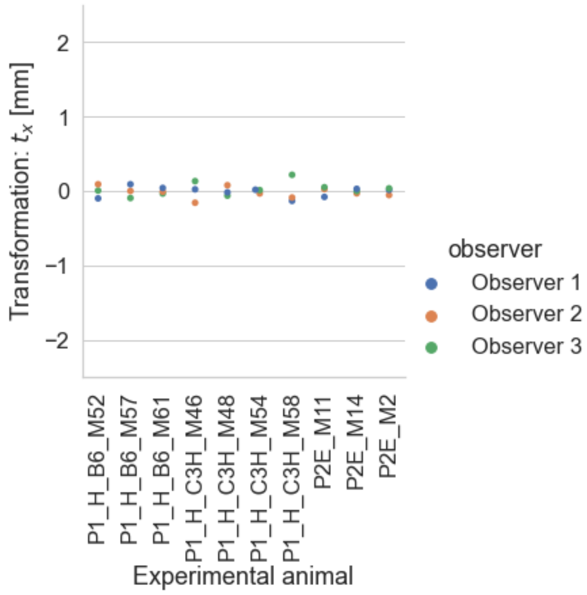

(a)

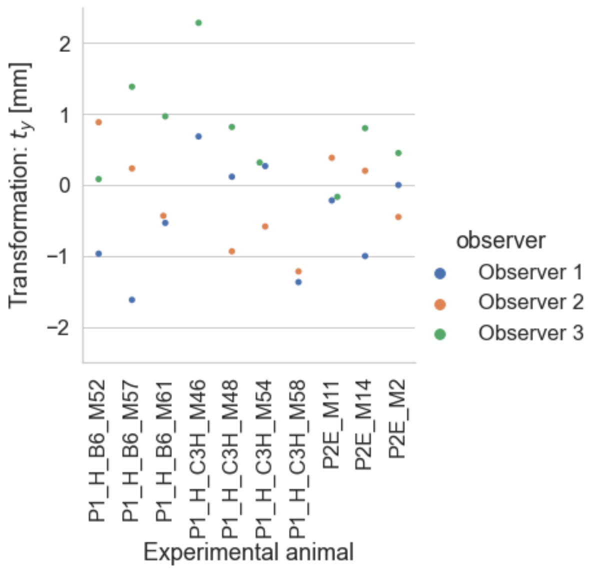

(b)

Figure S7: User-determined translation vector $\vec{t}$ in x (a) and y (b).

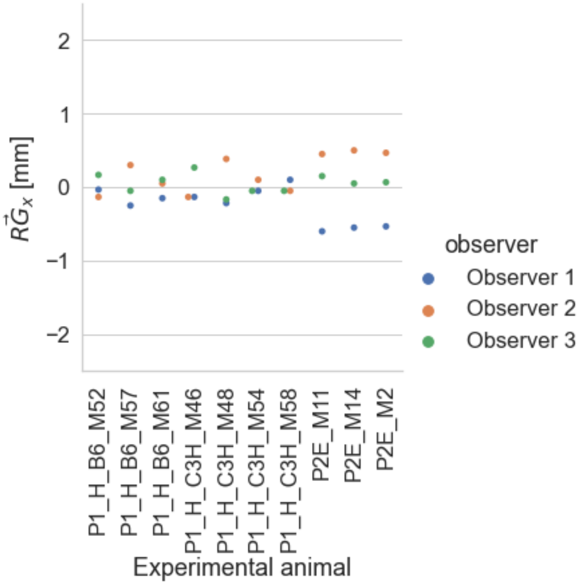

(a)

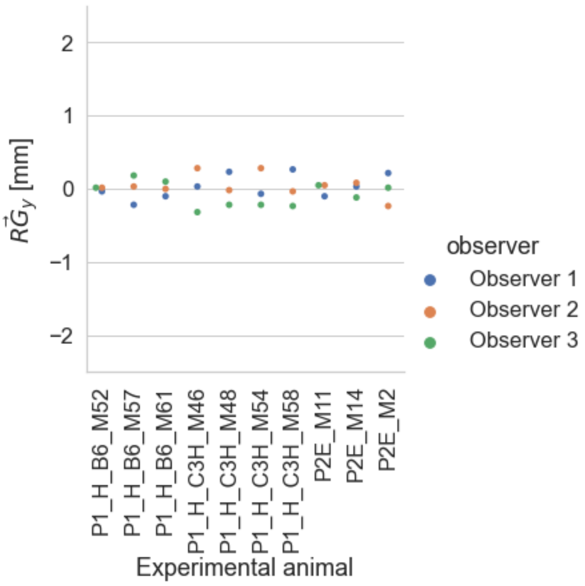

(b)

Figure S8: User-determined target coordinates $\vec{RG}$ as passed to the motorized stages vectors in x (a) and y (b).

3.2.2 Intra-observer variations

This section lists intra-observer variations for the above-mentioned parameters $\vec{XR}$, s, α, $\vec{t}$ and $\vec{RG}$ separately in x and y, where applicable. The x- and y-coordinates refer to the horizontal and vertical axes in beam's eye view. Similar to the inter-observer variations, we normalized the resulting values for $\vec{XR}$, α, $\vec{t}$ and $\vec{RG}$ to the animal-wise mean for better visual comparison.

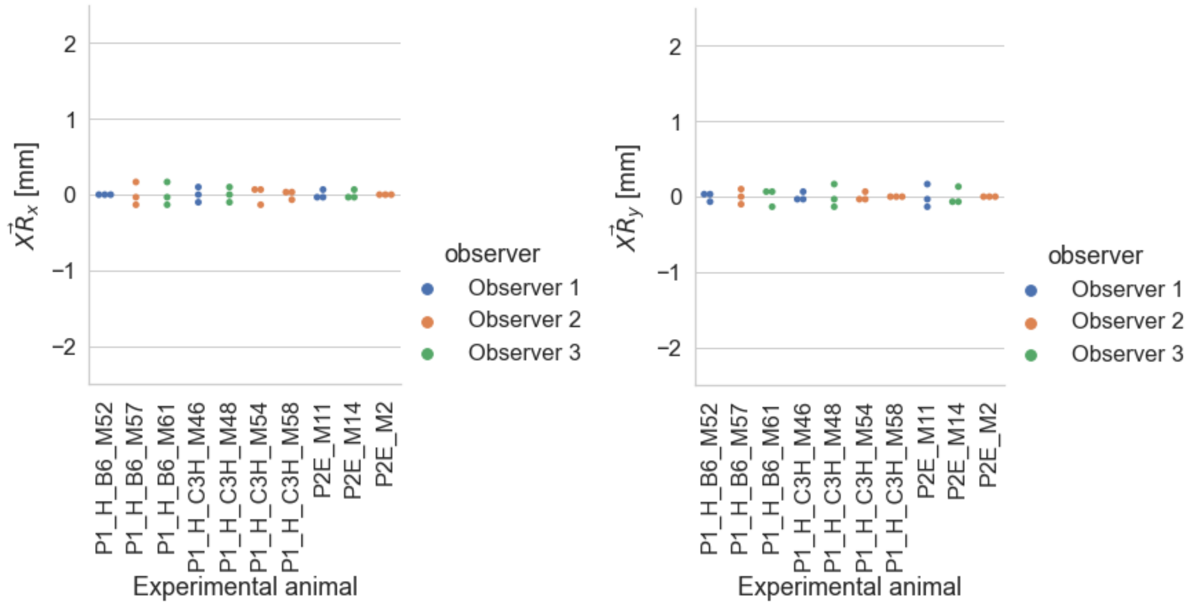

(a) (b)

Figure S9: User-determined coordinates $\vec{XR}$ in x (a) and y (b) in the CT-based coordinate system.

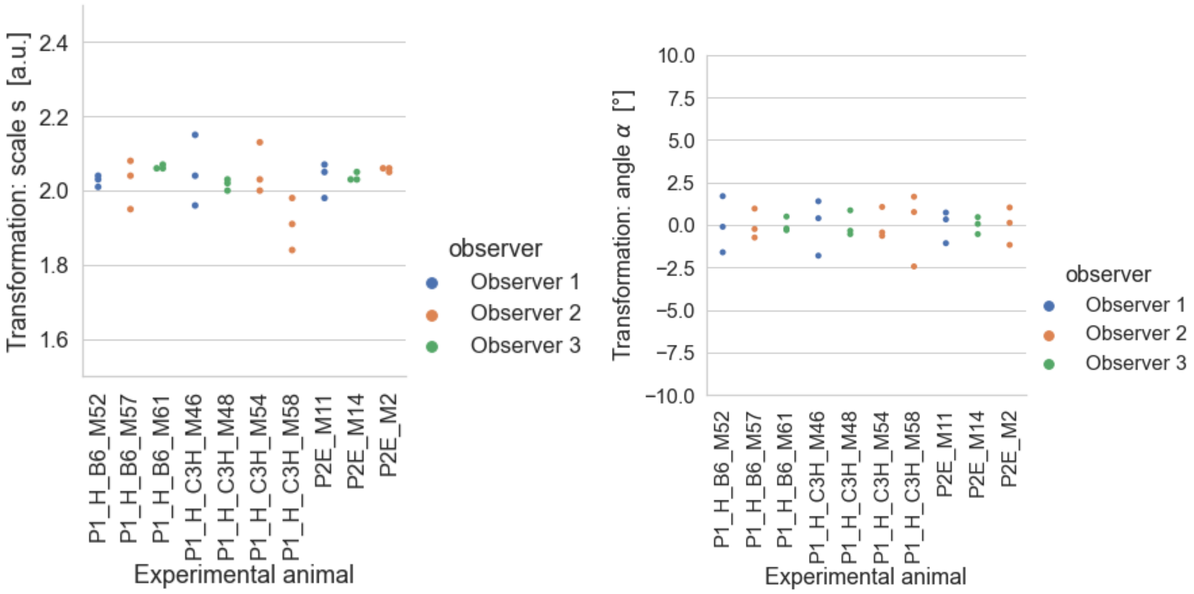

(a) (b)

Figure S10: User-determined scale s (a) and rotation angle α (b).

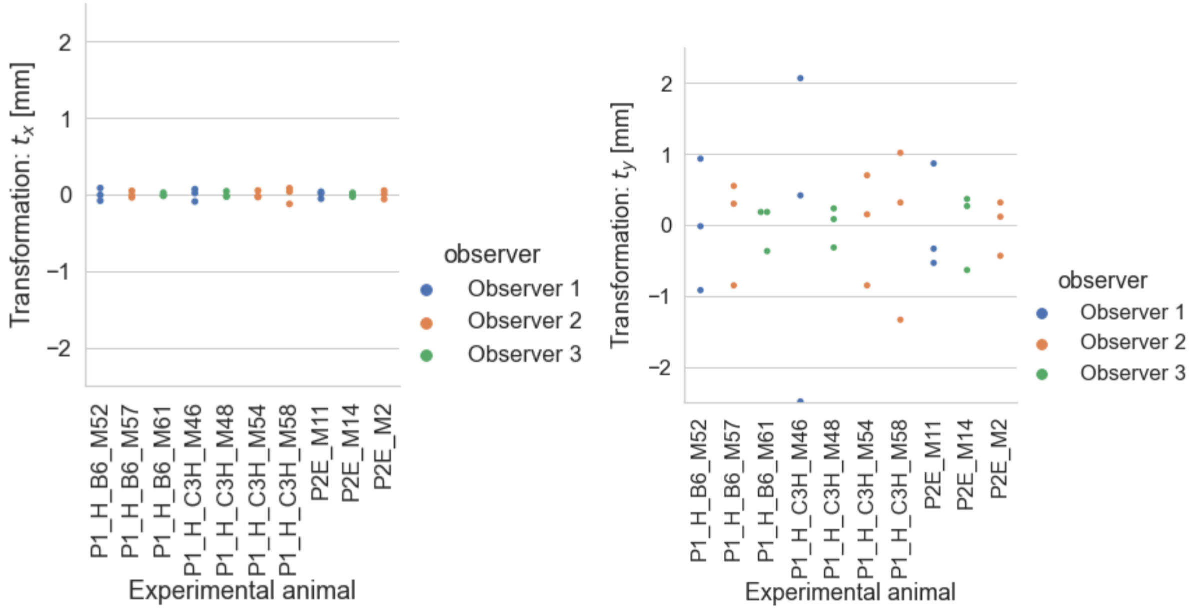

(a) (b)

Figure S11: User-determined translation vector $\vec{t}$ in x (a) and y (b).

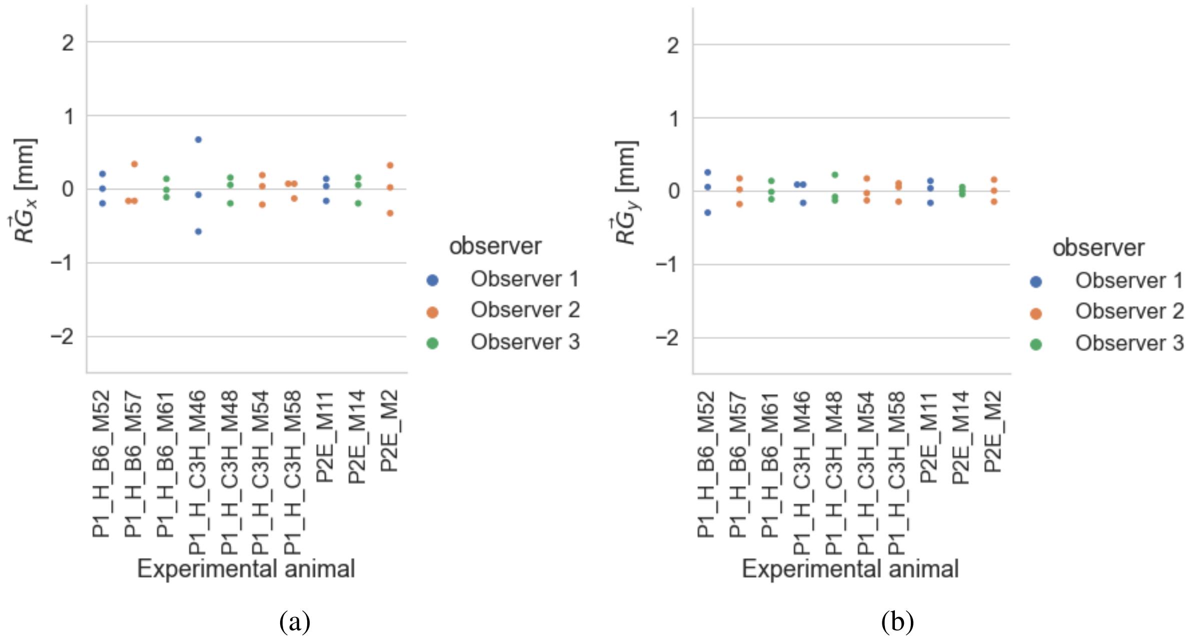

Figure S12: User-determined target coordinates $\vec{RG}$ as passed to the motorized stages vectors in x (a) and y (b).
